# Epithelial to Mesenchymal Transition in a Clinical Perspective

**DOI:** 10.1155/2015/792182

**Published:** 2015-09-06

**Authors:** Jennifer Pasquier, Nadine Abu-Kaoud, Haya Al Thani, Arash Rafii

**Affiliations:** ^1^Stem Cell and Microenvironment Laboratory, Department of Genetic Medicine and Obstetrics and Gynecology, Weill Cornell Medical College in Qatar, Education City, Qatar Foundation, P.O. Box 24144, Doha, Qatar; ^2^Department of Genetic Medicine, Weill Cornell Medical College, New York, NY 10021, USA

## Abstract

Tumor growth and metastatic dissemination rely on cellular plasticity. Among the different phenotypes acquired by cancer cells, epithelial to mesenchymal transition (EMT) has been extensively illustrated. Indeed, this transition allows an epithelial polarized cell to acquire a more mesenchymal phenotype with increased mobility and invasiveness. The role of EMT is quite clear during developmental stage. In the neoplastic context in many tumors EMT has been associated with a more aggressive tumor phenotype including local invasion and distant metastasis. EMT allows the cell to invade surrounding tissues and survive in the general circulation and through a stem cell phenotype grown in the host organ. The molecular pathways underlying EMT have also been clearly defined and their description is beyond the scope of this review. Here we will summarize and analyze the attempts made to block EMT in the therapeutic context. Indeed, till today, most of the studies are made in animal models. Few clinical trials are ongoing with no obvious benefits of EMT inhibitors yet. We point out the limitations of EMT targeting such tumor heterogeneity or the dynamics of EMT during disease progression.

## 1. Introduction

Despite the improvement of treatment regimens, cancer remains a leading cause of death worldwide. Metastatic disease is responsible for the majority of cancer-induced mortality [1]. The development of new therapeutic strategies targeting key factors driving metastasis remains a challenging goal for both clinicians and scientists. Metastasis is artificially divided into a series of sequential highly organized and organ specific steps [2]. Among these steps is the acquisition of migratory and invasive proprieties by cancer cells, which can be achieved through epithelial-mesenchymal transition (EMT) [3–6].

First described in embryogenesis, EMT is a cellular reprogramming process in which epithelial cells acquire a mesenchymal phenotype [7]. During this transformation, epithelial cells lose their polygonal shape and ability to grow in colonies, but they acquire spindle-shaped morphology and exhibit a more motile and invasive behavior [8]. These phenotypic changes are associated with proteins and gene modifications in different interconnected families such as transcription factors, cadherins, catenins, matrix metalloproteases (MMPs), or growth receptors [9, 10].

While EMT has been well accepted and demonstrated* in vivo* during embryogenesis, its implication in the metastatic process is still debated [11–16]. Identifying the EMT process in neoplastic disease is difficult since cells undergoing EMT share many molecular and morphological characteristics with the surrounding stromal fibroblasts. Moreover, although primary carcinoma or circulating tumor cells (CTCs) display EMT features, cells present in the distant metastases site are generally epithelial [17]. In 2002, Their proposed an explanation to such observation by describing the reversible EMT metastasis model in which primary epithelial tumor cells activate EMT to invade distant sites, and, upon arriving, they undergo a MET (mesenchymal-epithelial transition) to form an epithelial metastatic lesion [18].

Numerous reviews have comprehensively described EMT in cancer as well as the molecular pathways implicated in EMT or MET [17, 19–21]. The description of such findings is beyond the scope of this review. Here, we focus on the latest research on EMT in the clinical context for prognostic or therapeutic or strategies.

## 2. Can We Use EMT to Predict Patient's Outcome?

Recently, the detection of circulating tumor cells above a defined cut-off has been associated with poor prognosis in different cancers such as breast or prostate tumors [22, 23]. Circulating tumor cells, as well as metastatic lesions, of many different cancers present EMT characteristic [24–30]. Many studies investigated whether the expression of EMT markers would be associated with poor patient prognosis. The aberrant expression of Snail is related to poor patient survival in breast [31–34], ovarian [33, 35, 36], hepatocellular [37–40], and colorectal carcinomas [41, 42]. Twist overexpression is associated with a poor clinical outcome in many cancers such as bladder cancer [43], breast cancer [34], oral squamous cell carcinoma [44], ovarian cancer [45, 46], or cervical cancer [47]. Vimentin overexpression in cancers and its correlation with growth and metastasis suggest that it might be an indicator of poor prognostic for many cancers [48]. In bladder cancer, a study of eleven different cell lines revealed that the loss of E-cadherin expression is a marker of poor response to the monoclonal antibody cetuximab, which blocks EGFR binding [49]. More recently, Twist-1 promoter hypermethylation, studied on 65 surgically resected specimens, was shown to be a useful molecular marker for predicting prognosis and contralateral cervical lymph node metastases in patients with tonsillar squamous cell carcinoma [50].

The increasing amount of data on single EMT indicators urged the investigation of the correlation between several markers on patients' prognosis. A 4-EMT genes signature (E-cadherin (CDH1), inhibitor of DNA binding 2 (ID2), matrix metalloproteinase 9 (MMP9), and transcription factor 3 (TCF3)) was used to predict clinical outcome in a cohort of 128 hepatocellular carcinoma patients and then validated in an independent cohort of 231 patients with hepatocellular carcinoma from three different institutions [51]. The authors claimed that this 4-gene signature could improve patients' survival prediction on the risk score and tumor stage. Recently, in a study including surgical specimens from 78 cases of esophageal squamous cell carcinoma resected without preoperative treatment between 2001 and 2013, Niwa et al. demonstrated that the vimentin/E-cadherin ratio was correlated with tumor invasion and can serve as an independent prognostic factor among chemonaive patients [52]. In an analysis of 100 surgically resected hepatic tumors, EMT markers Twist-1 and Zeb-2 were shown to be involved in early disease recurrence in hepatocellular carcinoma and served as good prognosis markers [53]. In a different study on paraffin-embedded hepatocellular carcinoma tissues (*n* = 113) and their corresponding peritumoral normal tissues (*n* = 106), although the expression of 3-EMT-related proteins, S100A4, vimentin, and E-cadherin was studied, the authors reported that E-cadherin alone can be used as a direct prognosticator of negative outcome [54]. The prognostic significance of E-cadherin, twist, and vimentin was assayed in 121 patients with bladder cancer and, in this study, only vimentin appears as an independent predictor for cancer progression and survival [55].

Cancer stem cells have emerged as a particular entity within tumor cell plasticity. They have the ability to reinitiate the tumor in serial engraftment assays; they are more resistant to treatment than the bulk of the tumor and their role in the occurrence of metastasis has been suggested in several studies [1, 56–58]. Their identification relies on functional proprieties (spheroid formation in 3D media, asymmetric division, and serial passages in NOD/SCID mice) and specific markers. Several authors have described an increase of tumor stemness when cancer cells undergo EMT, leading to the study of stem cell associated markers combined with EMT-related markers as prognosis indicators. Luo et al. studied the correlation of SOX2, OCT4, and Nanog with E-cadherin, N-cadherin, and Snail in a nasopharyngeal carcinoma cohort of 122 patients [59]. They demonstrated that OCT4 and Nanog could be used as poor prognosis factor and are linked with the progression of the invasive front. In two different clinical studies, one on 119 human cholangiocarcinoma patients [60] and one on 276 consecutive primary gastric cancers and 54 matched lymph node metastases [61], the same team revealed that EMT markers (Snail-1, Zeb-1, E-cadherin, vimentin, and beta-catenin) and the CSC marker, CD44, are strongly correlated. Moreover, they revealed that the simultaneous expression of Snail-1, vimentin, E-cadherin, and CD44 was associated with advanced stage, metastasis, and invasion and was an independent indicator for disease-free survival.

Accumulating evidences in the literature are reflecting the regulatory roles of microRNAs (miRNAs) on EMT phenotype [62]. miRNAs can be optimal markers of specific disease or a patient' prognosis. Indeed, they can be found and quantified in many different biological fluids, including blood, urine, and cerebrospinal fluid; they also display great stability (even after boiling, freeze-thaw cycles, or low or high pH conditions). For instance, tumors with low expression of miR-335 and miR-126, 2 miRNA known to inhibit the first step of EMT, have been reported to present more probability to develop metastasis than tumors with higher expression of these miRNA [63]. In the blood, qPCR analysis of miR-10b, miR-34, and miR-155 allows the discrimination between patients with breast cancer metastasis and healthy controls. These miRNA play an important role in regulating EMT in response to TGF*β*. A meta-analysis of 17 studies with various carcinomas uncovered the role of miR-21 (an oncomiR known to promote EMT through TGF*β* pathway) as a poor prognosis biomarker in breast, squamous cell carcinoma, astrocytoma, and gastric cancer [64].

As described above, many EMT markers or their derivatives have been associated with patients' prognosis in different studies. However, while an EMT phenotype seems clearly associated with an increased metastatic phenotype, the use of such markers has not yet been translated into clinical practice. The requirements for an assay to be usable in clinical practice are quite stringent. Indeed, any prognosis marker has to first display great robustness. It should be reproducible among different laboratories and between pathologists. As prognostic markers will be used to give adjuvant therapy, their specificity should be quite high allowing the identification of patients who would not benefit from adjuvant treatment. While EMT markers have been independently associated with patients' prognosis, they have not yet been used in clinical practice for several reasons.

Tumor heterogeneity is one of the main reasons; the expression of EMT markers can vary at different locations of a tumor (usually increased mesenchymal phenotype at the periphery). Clear cut-offs are critical; most of the markers used in classical pathology have clear cut-offs (mitosis per field, Her2 overexpression, etc.). For EMT markers, defining the cut-offs might be difficult as different tumors display different levels of epithelial or mesenchymal phenotype and it is hard to clearly attribute a value for a particular marker. These are few difficulties among others including technical issues to do multiplex assays and the lack of large multicentric prospective trials.

One solution might be the advent of oncogenomic prognosis assays based on gene expression [65]. Currently, more than 100 clinical trials in cancer disease are ongoing using EMT as a keyword. Most of them study the prevalence of EMT markers in different cancers and their potential use as prognosis factor. The data from these studies will help us determine the clinical context where EMT could be used to tailor patients' treatment.

## 3. Can We Use EMT Effectors to Treat Patients?

EMT is an extremely well-organized process, activated in response to a combination of extracellular cues from the tumor microenvironment. EMT-inducing signals seem to be cell or tissue specific and require the cooperation between multiple signaling pathways and regulators. We considered the potential targets in three different groups classified based on their role during EMT: the molecular effectors executing EMT, the transcription factors acting as regulators to orchestrate EMT, and extracellular inducers that engage the cells in EMT.

### 3.1. Effectors

EMT effectors are mostly proteins that define the epithelial or mesenchymal phenotype of a cell. A key feature of EMT is the switch from E-cadherin (marker of epithelial cells) to N-cadherin (makers of mesenchymal cells) [9]. Targeting these cadherins in order to avoid a loss of E-cadherin or an upregulation of N-cadherin could therefore be a promising strategy.

Several groups studied the transfection of E-cadherin in highly mesenchymal and invasive cells and showed a reversion of the poorly differentiated carcinoma into a well-differentiated one with a minimally invasive epithelial phenotype [66–70]. In breast cancer, it has been demonstrated that salinomycin can selectively kill E-cadherin-negative breast epithelial cells as compared with E-cadherin-positive cells in NOD/SCID and Balb/c mice model [71]. Global gene expression analyses of breast tissues isolated directly from patients display that salinomycin treatment results in the loss of cancer stem cell (CSC) expression. The tumor suppressor role of E-cadherin has been established in many cancers including hepatocellular carcinoma [72], esophagus [73], melanoma [74], breast cancer [75, 76], or squamous cell carcinoma of the skin, head, and neck [77, 78]. However, in ovarian tumors, E-cadherin is consistently upregulated and maintained in ovarian carcinoma cells that metastasize to the peritoneum and omentum [79]. E-cadherin expression has been found in patients with a family history of ovarian cancer, proposing a potential role of E-cadherin in tumor initiation and/or progression in this particular cancer [80]. Concordantly, several recent studies point to a promoting role of E-cadherin during tumor progression in different epithelial cancers such as ovarian, breast, or brain cancer (reviewed in [81]). An epithelial phenotype has been also correlated with an increase in cancer stemness and engraftment in host organs in prostate cancer [82]. Overall, targeting E-cadherin seems to be difficult due to its ambiguous role in carcinogenesis.

Inhibition of N-cadherin has been assessed in several studies [83, 84]. Shintani et al. reported that, in a mouse model of pancreatic cancer, the peptide ADH-1 is able to block N-cadherin and prevent tumor progression [85]. In head and neck cancer cell line, quercetin has been shown to significantly reduce the migration ability of sphere cells by decreasing N-cadherin production [86]. Recently, Sadler et al. demonstrated that targeting N-cadherin using a neutralizing antibody may be a good therapeutic strategy to treat multiple myeloma [87].

Vimentin is also a canonical marker of mesenchymal phenotype and therefore an important effector of EMT [48]. Few reports have shown a direct inhibition of vimentin. Lahat and collaborator suggested that the withaferin-A induces vimentin degradation in a panel of soft tissue sarcoma xenograft experiments, leading to the inhibition of growth, local recurrence, and metastasis [88]. In prostate tumors, both silibinin and flavonolignan inhibited invasion, motility, and migration of the cancer cells via downregulation of vimentin in cancer cell lines and mice models [89, 90]. Finally, salinomycin, an antibiotic, reduced significantly vimentin level and induced increase in E-cadherin expression in CD133^+^ colorectal cancer cell lines HT29 and SW480 resulting in decreased malignant traits [91].

None of these strategies are currently tested in a clinical context. Indeed, EMT effectors have a complex role and their function might be time and context dependent during the metastatic process such as illustrated by the dual role of E-cadherin. Some additional key EMT effector molecules are proteins that promote cell migration and invasion during the process such as fibronectin, PDGF/PDGF receptor autocrine loop, Cd44, or integrin *β*6 [17]. Hence, these proteins might also be considered as potential targets to counter the EMT process.

### 3.2. Regulators

EMT regulators are a core of transcription factor such as Snail-1/Snail-1, basic helix-loop-helix family (E47, E2-2, and Twist-1/Twist-2), and Zeb-1/Zeb-1 [21, 92]. The role of these transcription factors in proliferation, invasion, and migration of epithelial tumors has been well described and their use as a target to block EMT process seems appealing [41, 93–95].

da Silva et al. reported that the inhibition of Twist-1 in metastatic oral squamous cell carcinoma (OSCC) induced a potent inhibition of cell invasiveness* in vitro* as well as* in vivo* using an orthotropic mouse model of metastatic OSCC [44]. The secreted frizzled-related protein (sFRP1 and sFRP2) two Wnt antagonists enhance the expression of E-cadherin through the inhibition of Twist-1 and suppress the invasiveness of cervical cancer* in vivo* in a xenograft animal model [96]. The bone morphogenetic protein 7 (BMP7) is a potential metastasis inhibitor that disrupts EMT through Twist-1 inhibition in melanoma WM-266-4 and HEK293T cell lines [97]. Arumugam et al. have shown using many different pancreatic cell lines that silencing Zeb-1 not only restored the expression of epithelial marker genes, but also increased cellular sensitivity to therapeutic reagents [98]. In a lung carcinoma cell line, the knockdown of Snail or Twist-1 is able to restore the cell chemosensitivity to cisplatin [99, 100].

The sulforaphane, an organosulfur compound, is able to downregulate Twist-1 as well as other EMT proteins like vimentin leading to a decrease of stemness properties in PANC-1, MIA PaCa-2, AsPC-1, and Bx PC-3 pancreatic cells lines [101]. Recently, moscatilin was shown to target the Akt-Twist-1 dependent pathway and decrease the migration and metastasis of MDA-MB-231 breast cancer cell line [102]. Fucoidan was also described to inhibit EMT in breast cancer cell lines such as 4T1 and MDA-MB-231 through the decreased Twist-1, Snail, and Slug expression [103]. In 2014, Myung and collaborators demonstrated that the knockdown of Snail with siRNA technique in three glioblastoma cell lines (KNS42, U87, and U373) suppresses the proliferation, viability, migration, and invasion of cells by disrupting the EMT process [104].

Despite the promising results in preclinical studies, overall, EMT core transcription factors remain technically challenging to target in a clinical setting. However, a clinical trial is currently investigating the molecular mechanism and clinical significance of the interplay between Twist-1 and other EMT regulators through microRNA-29 family in head and neck squamous cell carcinoma (NCT01927354).

### 3.3. Inducers

The principal inducers of EMT are proteins from the TGF*β* (TGF*β*1, TGF*β*2, TGF*β*3, inhibins, activin, anti-Müllerian hormone, bone morphogenetic protein, decapentaplegic, and Vg-1) and the growth factor (fgf, hgf, egf, and igf1) families [21]. High-throughput drug screening has been performed to identify potential inhibitors of EMT in response to various inducers. The majority of molecules selected inhibit specific EMT-inducing signals used in the screen. For instance, rapamycin and 17-AGG have been shown to inhibit TGF*β*-induced EMT through the modification of TGF*β* pathway itself as assessed by a global gene expression profile from a cell culture model of TGF*β*-induced EMT [105]. Inhibitors of ALK5, MEK, and SRC are able to block EMT in response to EGF, HGF, and IGF-1 [105, 106]. In 2011, two different groups proposed c-MET as a potential therapeutic target in hepatocellular carcinoma cell lines Huh7, Hep3B, MHCC97-L, and MHCC97-H [107] and BNL CL.2 (BNL) and BNL 1ME A. 7R.1 (1MEA) [108]. In prostate cancer, inhibition of c-Met expression and Met-mediated signaling by Frzb leads to the upregulation of epithelial markers and a decrease of the mesenchymal traits in a xenograft mouse model [109].

The most studied inducer of EMT remains TGF*β* [110–112]. Targeting TGF*β* pathway to alter EMT induced tumor cell invasion may be appropriate as metastasis prevention strategies in early stage carcinomas. SD-093 and LY-580276, two competitive inhibitors for the ATP-binding site of TGF*β*RI kinase, disrupt EMT and tumor cell migration in many cancers [113, 114]. EW-7203, EW-7195, and EW-7197, specific TGF*β*/ALK5 inhibitors available as orally administered drugs [115, 116], have been shown to inhibit EMT in both TGF*β* treated breast cancer cells and 4T1 orthotropic xenograft mice [117]. Bone morphogenetic protein 7 (BMP7) was revealed as a potential inhibitor of EMT induced by TGF*β* in thirty liver tissue samples of patients with cholangiocarcinoma [118].

Currently, the only compounds interfering with EMT in clinical trial are the ones able to block EMT inducers. In 2008, already, the LY2157299, a clinical selective TGF*β*1 receptor inhibitor, was undergoing a still unpublished phase I trial for colon, prostate, and adrenocortical or breast cancer and malignant melanoma patients [119]. That same year, another preclinical trial using human xenografts Calu6 (non-small-cell lung cancer) and MX1 (breast cancer) implanted subcutaneously in nude mice demonstrated that LY2157299 is able to reduce the tumor growth [120]. LY2157299 is now known to display antitumor effects in patients with glioblastoma and hepatocellular carcinoma [121]. LY2157299 is currently tested in four clinical trials, in patients recruiting state: Phase Ib/II in stages II–IV pancreatic cancer of LY2157299 combined with gemcitabine versus gemcitabine plus placebo (NCT01373164); Phase II in HCC patients with disease progression on sorafenib or who are not eligible to receive sorafenib (NCT01246986); Phase Ib/IIa study combining LY2157299 with standard temozolomide based radiochemotherapy in patients with newly diagnosed malignant glioma (NCT01220271); and Phase II Study of LY2157299 monotherapy or LY2157299 plus Lomustine therapy compared to Lomustine monotherapy in patients with recurrent glioblastoma (NCT01582269).

Erlotinib, an EGF receptor tyrosine kinase inhibitor, is approved for the treatment of second- and third-line advanced non-small-cell lung cancers [122, 123]. Interestingly, its efficacy is correlated with the EMT status of the cells; higher E-cadherin levels indicate sensitivity, whereas higher vimentin and Zeb-1 levels indicate resistance. Thus, in 2012, a randomized phase II trial on 132 patients with non-small-cell lung cancer evaluated the effect of erlotinib combined with the isoform selective histone deacetylase inhibitors, entinostat, known to prevent the resistance by reverting the cancer cell mesenchymal phenotype to an epithelial one [124]. Even if entinostat failed at improving the outcome of patients, the study revealed that E-cadherin expression levels at time of diagnosis could portray the sensitivity to HDACi/EGFR-TKI inhibition providing the basis for a biomarker-driven validation.

In 2014, the GC1008 (fresolimumab), a human anti-TGF*β* monoclonal antibody, has undergone a phase I clinical trial in patients with advanced malignant melanoma or renal cell carcinoma [125]. 29 patients, 28 with malignant melanoma and 1 with renal cell carcinoma, were included and received intravenous GC1008 at 0.1, 0.3, 1, 3, 10, or 15 mg/kg on days 0, 28, 42, and 56. It demonstrated that GC1008 was presenting an acceptable safety and toxicity, and the maximum dose, 15 mg/kg, was determined. Multiple doses of GC1008 demonstrated only preliminary evidence of antitumor activity, allowing further studies of single agent and combination treatments in clinical trials. In fact, GC-1008 is currently tested in two clinical studies: fresolimumab and radiotherapy in metastatic breast cancer (NCT01401062) and safety and imaging study of GC1008 in glioma (NCT01472731).

## 4. A Moving Target

Despite the massive amount of preclinical data in most cancer types, there is still no clear cancer treatment specifically targeting EMT. Many aspects of tumor biology can explain the gap between the preclinical and clinical data.

In most preclinical studies an induced model of EMT is used to demonstrate the implication of EMT in tumor spread and then to demonstrate the efficacy of targeting EMT in achieving tumor response. These models are usually quite far from clinical reality. They do not perfectly reproduce tumor heterogeneity which is increased at the metastatic stage as demonstrated by several studies of tumor phylogeny [126]. Such heterogeneity can be the result of cell intrinsic genomic differences or interaction with the microenvironment [127–133]. Tumor progression acquired through EMT can be transient and represent only a small timeframe in a patient's disease. Hence, targeting EMT will not have similar effect as in preclinical studies where EMT is constitutively activated. Many of the factors have dual or ambivalent roles. The recent demonstration of the role of MET in the establishment of metastasis in the host organ and its relationship to stemness in specific tumors adds a new degree of complexity to anti-EMT strategies. One could somehow potentially increase cancer stemness and improve host organ homing by inhibiting EMT.

## 5. Conclusion

**Figure 1:**
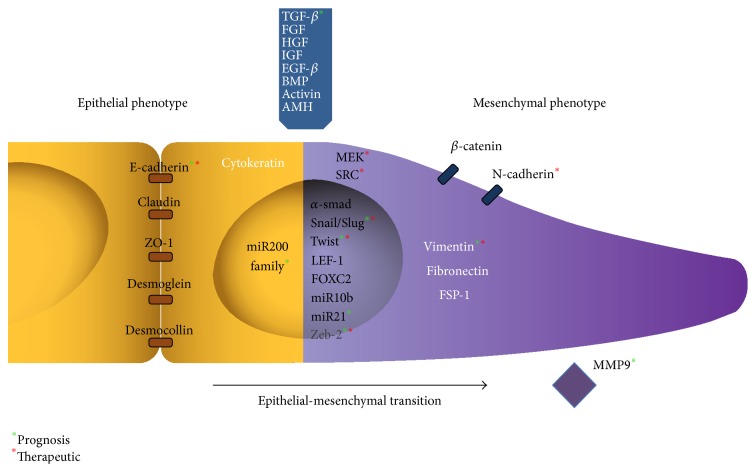
Epithelial to mesenchymal transition with effectors and inducers studied in the prognostic or therapeutic context. Green asterisk: implicated in prognosis. Red asterisk: targeted therapeutically.

Overall, despite the tremendous amount of preclinical data on the implication of EMT in cancer progression, there is still no routine clinical translation at both prognosis and therapeutic levels (Figure 1). Here, while we point out the different elements of the EMT cascades that could be targeted, we also underline the difficulties to translate the preclinical findings in routine clinic. However, we can hypothesize that as we enter the era of precision and personalized medicine, new technologies (next gene sequencing, circulating tumor cells, circulating tumor DNA, etc.) will help us better define patients' specific disease at precise time points during disease evolution. Such studies might then really illustrate whether EMT has a role in neoplastic evolution and point out the appropriate therapeutic window where EMT inhibition could lead to improved survival in patients.
